# Variants in the *CXCL12* gene was associated with coronary artery disease susceptibility in Chinese Han population

**DOI:** 10.18632/oncotarget.17171

**Published:** 2017-04-18

**Authors:** Junbo Zhang, Huijun Ma, Jie Gao, Shu Kong, Jiangtao You, Ying Sheng

**Affiliations:** ^1^ Department of Peripheral Vascular, First Affiliated Hospital of Xi’an Jiaotong University, Xi’an 710061, China; ^2^ Department of Cardiology, The First Hospital of Xi’an, Xi’an 710002, China; ^3^ Cadre Ward of the Second Affiliated Hospital of Xi’an Jiaotong University, Xi’an 710004, China; ^4^ Institute of Medical Engineering, Medical School of Xi’an Jiaotong University, Xi’an 710061, China; ^5^ Department of Thoracic Surgery, First Affiliated Hospital of Xi’an Jiaotong University, Xi’an 710061, China

**Keywords:** CXCL12, coronary heart disease, gene polymorphisms, case-control study

## Abstract

**Background:**

Coronary artery disease (CAD) is one of the most serious diseases all around the world. Previous studies have shown the function of *CXCL12* in the process of atherosclerosis. The aim of this research is to examine whether variants of *CXCL12* contribute to CAD.

**Materials and Methods:**

To examine whether variants of *CXCL12* contribute to CAD, we selected 6 single nucleotide polymorphisms (SNPs) of *CXCL12*, and genotyped by Sequenom MassARRAY technology in 597 CAD patients and 685 healthy control. Odds ratio (OR) and 95% confidence intervals (CIs) were calculated by unconditional logistic regression adjusted for age and gender. We also analysis the differences in continuous variables among the subjects with three genotypes of related genes were assessed using the ANOVA.

**Results:**

We found significant differences in apoB concentrations with rs1065297 and rs10793538 different genotype. In the allele model, rs1065297, rs266089 and rs10793538 in *CXCL12* gene associated with the risk of CAD. Stratified according to gender, rs266089 and rs2839693 in *CXCL12* gene were associated with the risk of CAD in men, while rs1065297 and rs10793538 in *CXCL12* gene were associated with the risk of CAD in women. Stratified according to age, rs197452 decreased the risk of CAD in less than 50 years old group. While in more than 50 years old group, not find significant results. Haplotype analysis shown that haplotype “TGCC” in the block increased CAD risk (OR=1.26, 95%CI: 1.00-1.58, *p*=0.046).

**Conclusion:**

This study provides an evidence for polymorphism of *CXCL12* gene associated with CAD development in Chinese Han population.

## INTRODUCTION

Coronary artery disease (CAD), also named as coronary heart disease (CHD) or ischaemic heart disease (IHD), has become one of the most common cause of morbidity and mortality in cardiovascular diseases (CVDs) all around the world, especially in the industrial countries [1]. Coronary heart disease (CHD) is caused by obstruction of epicardial coronary artery that supplies blood and oxygen to the heart. Multiple factors, such as lifestyle and environmental factors, play critical roles in the occurrence and progression of CHD. The characteristic pathological changes of CAD is atherosclerosis that results from the endothelial injury or functional disorder, which is triggered by the abnormal accumulation of lipoproteins in the intima [2]. Plenty of cells such as T cells, dendritic cells, neutrophils and macrophages are also involved into the process of the disease. Traditional environmental risk factor for CAD contains tobacco smoking, hypertension, diabetes or hyperglycemia, hyperlipidemia especially high levels of LDL and obesity or overweight [3]. There is a growing worldwide awareness of that heritable factors play an important role in the progress of atherosclerosis [4] and relatively a great many genome-wide association studies (GWAS) have confirmed the effect of genes in CAD [5–7].

Atherosclerosis is actually a chronic inflammatory reaction in blood vessels and includes two remarkable steps, foam cell formation and smooth muscle cell recruitment. Chemokine (C-X-C motif) ligand 12 (CXCL12), also called stromal cell-derived factor-1 (SDF-1), is one of the members of CXC chemokine family [8] and mostly known for its crucial role in the accumulation of smooth muscle progenitor cells (SPCs) [9]. It will trigger MAPK and PI3K signal pathway [10] by binding to a G protein-coupled receptor, CXCR4, and contribute to macrophage migration which results from lipid accumulation especially low-density lipoprotein (LDL) [11]. Additionally, it is identified that different single nucleotide polymorphisms (SNPs) of *CXCL12* participates in various disease such as neck and head squamous cell carcinoma [12], myelodysplastic syndromes [13], multiple sclerosis [14], etc. Here, we choose six SNPs (rs1065297, rs1801157, rs266089, rs197452, rs2839693 and rs10793538) of *CXCL12* to explore which SNPs are associated with the risk of CAD.

## RESULTS

After all the experiments and data compilation, 597 cases (376 males and 221 females) and 685 controls (385 males and 300 females) were included in the final analysis. As listed in Table 1, the mean age of cases is 61.44 while the control group is 48.59. The p value of age and sex were less than 0.001 and 0.014, respectively. Clinical and biochemical index of information in CAD patients in the Table 1, including ALT, AST, GGT, TP, GLU, TG, TC, HDL, LDL, apoA, apoB, LP(a), ect.

**Table 1:** Basic information of case and control groups

	Case		Control		*p*
	female	male	female	male	
<55	41	124	214	184	
≥55	180	252	86	101	
total	597		685		0.014
Mean age	61.44		48.59		*p* <0.001
ALT (U/L)	31.15±2.11				
AST (U/L)	36.41±2.93				
GGT (U/L)	44.65±3.77				
TP(g/L)	66.39±0.3				
GLU (mmol/L)	6.34±0.11				
TG (mmol/L)	1.8±0.07				
TC (mmol/L)	4.09±0.06				
HDL-C (mmol/L)	1.13±0.01				
LDL-C (mmol/L)	1.92±0.04				
APOA1 (g/L)	1.26±0.01				
APOB (g/L)	1±0.02				
Lp(a) (mg/L)	239.2±12.14				

ALT: alanine aminotransferase, AST: aspartate aminotransferase, GGT: gamma-glutamyl transpeptidase, TP: total protein, GLU: glucose, TG: triglyceride, TC: total cholesterol, HDL: high-density lipoprotein, LDL: low-density lipoprotein, apoA: apolipoprotein A, apoB: apolipoprotein B, LP(a): lipoprotein.

*p* <0.05, statistical significance.

The basic characteristics of the study subjects stratified by different genotype are shown in Table 2. We found significant differences in apoB concentrations with rs1065297 and rs10793538 different genotype. For rs1065297, the mean serum apoB concentration was 1.03 for the GG genotype (highest), 0.76 for the GA genotype (lowest), 1.01 for AA genotype (*p* = 0.003). For rs10793538, the mean serum apoB concentration of TT, TA and AA is 1.03, 0.76, and 1.01 respectively.

**Table 2:** Characteristics of the subjects with 3 different genotypes

SNP		Genotype			p
rs1065297		GG	GA	AA	
LDL-C	sample size	1	27	402	
	Mean±std	2.43	1.53±0.65	1.94±0.84	0.036
apoB	sample size	1	27	401	
	Mean±std	1.03	0.76±0.31	1.01±0.37	0.003
rs1801157		CC	CT	TT	
TP	sample size	272	148	22	
	Mean±std	67.03±6.33	65.44±6.26	64.83±6.47	0.023
rs10793538		TT	TA	AA	
apoB	sample size	1	26	402	
	Mean±std	1.03	0.76±032	1.01±0.37	0.004

*p* <0.05, statistical significance.

The allele information including MAF, HWE, OR etc. were demonstrated in Table 3. In the allele model, rs1065297, rs266089 and rs10793538 in *CXCL12* gene associated with the risk of CAD. Among them *CXCL12* rs1065297 and rs10793538 decreased CAD risk (rs1065297: OR=0.64, 95%CI: 0.46-0.90, *p*=0.010; rs10793538: OR=0.67, 95%CI: 0.47-0.93, *p*=0.016). Rs266089 in *CXCL12* gene increased the risk of CAD (OR=1.23, 95%CI: 1.00-1.51, *p*=0.046). Stratified according to gender, in the allele model, rs266089 and rs2839693 in *CXCL12* gene were associated with the risk of CAD in men, while rs1065297 and rs10793538 in *CXCL12* gene were associated with the risk of CAD in women.

**Table 3:** Basic SNPs in *CXCL12* gene summary of all study participants

SNP	Base/Change	MAF		HWE*p*	Total		Male		Female	
		Case	Control		OR(95%CI)	*p*	OR(95%CI)	*p*	OR(95%CI)	*p*
rs1065297	G/A	0.046	0.07	0.04	0.64(0.46-0.90)	0.01	0.74(0.48-1.14)	0.166	0.51(0.29-0.89)	0.017
rs1801157	T/C	0.217	0.21	0.644	1.04(0.86-1.26)	0.655	1(0.78-1.27)	0.999	1.1(0.81-1.49)	0.54
rs266089	A/G	0.19	0.16	0.201	1.23(1.00-1.51)	0.046	1.46(1.11-1.91)	0.006	0.99(0.72-1.36)	0.938
rs197452	T/C	0.116	0.116	0.852	1.00(0.78-1.27)	0.97	0.96(0.7-1.31)	0.781	1.05(0.72-1.53)	0.808
rs2839693	T/C	0.168	0.145	0.642	1.19(0.96-1.47)	0.113	1.44(1.09-1.89)	0.01	0.89(0.63-1.26)	0.529
rs10793538	T/A	0.046	0.068	0.119	0.669(0.47-0.93)	0.016	0.77(0.5-1.2)	0.249	0.52(0.3-0.92)	0.019

*p* <0.01, statistical significance for HWE

*p* <0.05, statistical significance.

We further explored the relationship between *CXCL12* gene and CAD in four genetic models (genotype, dominant, recessive and log-additive model) by unconditional logistic regression (Table 4). Results show that the odds of having CAD would be 1.30-fold (95%CI: 1.30-1.65, *p*=0.028) with GA-AA genotype, compared with the subjects with the homozygous GG genotype in the dominant model. A log-additive model revealed a potential association with CAD (OR = 0.67, 95%CI: 0.48-0.94, *p*= 0.018). But after adjusting by gender and age, we found no association between the SNPs and CAD.

**Table 4:** Associations between *CXCL12* SNPs and CAD

SNP	Model	Genotype	control	case	Crude analysis		adjusted by age and gender	
					OR (95% CI)	*p*-value	OR (95% CI)	*p*-value
rs266089	Codominant	G/G	487 (71.2%)	391 (65.5%)	1	0.086	1	0.18
		G/A	175 (25.6%)	185 (31%)	1.32 (1.03-1.68)		1.31(0.97-1.75)	
		A/A	22 (3.2%)	21 (3.5%)	1.19 (0.64-2.19)		0.91(0.43-1.94)	
	Dominant	G/G	487 (71.2%)	391 (65.5%)	1	**0.028**	1	0.11
		G/A-A/A	197 (28.8%)	206 (34.5%)	1.30 (1.03-1.65)		1.26(0.95-1.67)	
	Recessive	G/G-G/A	662 (96.8%)	576 (96.5%)	1	0.77	1	0.65
		A/A	22 (3.2%)	21 (3.5%)	1.10 (0.60-2.02)		0.84(0.40-1.78)	
	Log-additive	---	---	---	1.23 (1.00-1.50)	0.048	1.17(0.91-1.49)	0.22
rs10793538	Codominant	A/A	594 (87.2%)	544 (91.1%)	1	0.06	1	0.61
		T/A	81 (11.9%)	51 (8.5%)	0.69 (0.48-0.99)		0.91(0.58-1.41)	
		T/T	6 (0.9%)	2 (0.3%)	0.36 (0.07-1.81)		0.40(0.05-3.21)	
	Dominant	A/A	594 (87.2%)	544 (91.1%)	1	**0.025**	1	0.54
		T/A-T/T	87 (12.8%)	53 (8.9%)	0.67 (0.46-0.95)		0.87(0.57-1.35)	
	Recessive	A/A-T/A	675 (99.1%)	595 (99.7%)	1	0.2	1	0.37
		T/T	6 (0.9%)	2 (0.3%)	0.38 (0.08-1.88)		0.40(0.05-3.25)	
	Log-additive	---	---	---	0.67 (0.48-0.94)	**0.018**	0.86(0.57-1.28)	0.45

*p* <0.05, statistical significance.

Stratified according to gender, we found that under the dominant model rs266089 “GA-AA” genotype and rs2839693 “CT-TT” genotype increased the risk of CAD in men (rs266089: OR=1.54, 95%CI: 1.13-2.10, *p*=0.006; rs2839693: OR=1.51, 95%CI: 1.10-2.07, *p*=0.010) (Table 5). While in the women, rs1065297 and rs10793538 decreased the CAD risk under the log-additive model (rs1065297: OR=0.52, 95%CI: 0.30-0.91, *p*=0.021; rs10793538: OR=0.53, 95%CI: 0.31-0.93, *p*=0.027) (Table 5).

**Table 5:** The relationship between *CXCL12* genetic polymorphism and CAD was analyzed according to the gender stratification

	SNP	Model	Genotype	control	case	Crude analysis		adjusted by age and gender	
						OR (95% CI)	p-value	OR (95% CI)	p-value
Male	rs266089	Genotype	GG	284	242	1	0.022	1	0.054
			GA	92	119	1.52(1.10-2.10)	0.011	1.57(1.09-2.28)	0.017
			AA	10	18	1.76(0.78-3.99)	0.176	1.34(0.51-3.50)	0.551
		Dominant	GG	284	242	1	0.006	1	0.016
			GA-AA	102	137	1.54(1.13-2.10)		1.55(1.08-2.21)	
		Recessive	GG-GA	376	361	1	0.282	1	0.733
			AA	10	18	1.56(0.69-3.52)		1.18(0.45-3.07)	
		Log-additive	---	---	---	1.45(1.11-1.19)	0.007	1.41(1.04-1.93)	0.029
	rs2839693	Genotype	CC	292	252	1	0.036	1	0.19
			CT	86	111	1.50(1.01-2.08)	0.016	1.40(0.96-2.04)	
			TT	9	13	1.67(0.70-3.98)	0.244	1.45(0.52-4.08)	0.477
		Dominant	CC	292	252	1	0.01	1	0.069
			CT-TT	95	124	1.51(1.10-2.07)		1.40(0.97-2.02)	
		Recessive	CC-CT	378	363	1	0.353	1	0.584
			TT	9	13	1.50(0.64-3.56)		1.33(0.48-3.73)	
		Log-additive	---	---	---	1.42 (1.08-1.87)	0.012	1.33(0.97-1.83)	0.079
Female	rs1065297	Genotype	AA	257	203	1	0.17	1	0.614
			AG	40	18	0.57(0.32-1.02)	0.06	0.68(0.32-1.45)	0.323
			GG	3	0	/	/	/	/
		Dominant	AA	257	203	1	0.032	1	0.244
			AG-GG	43	18	0.53(0.3-0.95)		0.64(0.3-1.35)	
		Recessive	AA-AG	297	221	1	/	1	/
			GG	3	0	/		/	
		Log-additive	---	---	---	0.52(0.3-0.91)	0.021	0.63(0.31-1.28)	0.199
	rs10793538	Genotype	AA	258	203	1	0.206	1	0.787
			AT	39	18	0.59(0.33-1.06)	0.075	0.77(0.36-1.63)	0.489
			TT	3	0	/	/	/	/
		Dominant	AA	258	203	1	0.041	1	0.379
			AT-TT	42	18	0.54(0.3-0.97)		0.72(0.34-1.51)	
		Recessive	AA-AT	297	221	1	/	1	/
			TT	3	0	/		/	
		Log-additive	**---**	**---**	**---**	0.53(0.31-0.93)	0.027	0.69(0.34-1.4)	0.307

*p* <0.05, statistical significance.

Stratified according to age, we found that under the log-additive model, rs197452 decreased the risk of CAD in less than 50 years old group (rs197452: OR=0.63, 95%CI: 0.39-1.00, *p*=0.042). While in more than 50 years old group, we did not find significant results (Table 6).

**Table 6:** The relationship between *CXCL12* genetic polymorphism and CAD was analyzed according to the age stratification

	SNP	Model	Genotype	control	case	Crude analysis		adjusted by age and gender	
						OR (95% CI)	*p*-value	OR (95% CI)	*p*-value
<55	rs197452	Codominant	C/C	393 (78.9%)	141 (85.5%)	1	0.073	1	0.081
			C/T	101 (20.3%)	24 (14.6%)	0.66 (0.41-1.08)		0.68 (0.42-1.11)	
			T/T	4 (0.8%)	0 (0%)	0.00 (0.00-NA)		0.00 (0.00-NA)	
		Dominant	C/C	393 (78.9%)	141 (85.5%)	1	0.06	1	0.077
			C/T-T/T	105 (21.1%)	24 (14.6%)	0.64 (0.39-1.03)		0.65 (0.40-1.06)	
		Recessive	C/C-C/T	494 (99.2%)	165 (100%)	1	0.13	1	0.11
			T/T	4 (0.8%)	0 (0%)	0.00 (0.00-NA)		0.00 (0.00-NA)	
		Log-additive	---	---	---	0.63 (0.39-1.00)	**0.042**	0.64 (0.40-1.02)	0.053
≥55	rs197452	Codominant	C/C	141 (75.4%)	328 (75.9%)	1	0.97	1	0.96
			C/T	42 (22.5%)	94 (21.8%)	0.96 (0.64-1.46)		0.95 (0.63-1.44)	
			T/T	4 (2.1%)	10 (2.3%)	1.07 (0.33-3.48)		1.09 (0.33-3.53)	
		Dominant	C/C	141 (75.4%)	328 (75.9%)	1	0.89	1	0.86
			C/T-T/T	46 (24.6%)	104 (24.1%)	0.97 (0.65-1.45)		0.97 (0.65-1.44)	
		Recessive	C/C-C/T	183 (97.9%)	422 (97.7%)	1	0.89	1	0.88
			T/T	4 (2.1%)	10 (2.3%)	1.08 (0.34-3.50)		1.10 (0.34-3.55)	
		Log-additive	---	---	**---**	0.99 (0.69-1.40)	0.94	0.98 (0.69-1.39)	0.92

*p* <0.05, statistical significance.

**Figure 1:**
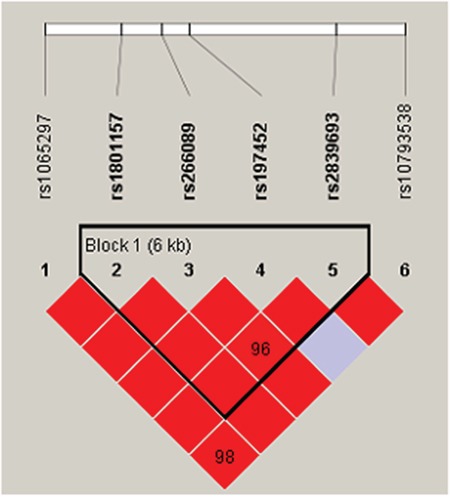
Haplotype block for the SNPs of CXCL12

Only one block was detected in the analysis using haploview software. The block consisted of rs1801157, rs266089, rs197452 and rs2839693 (Figure 1). The result of the association between *CXCL12* haplotype and CAD risk were listed in Table 7. We found that haplotype “TGCC” in the block increased CAD risk (OR=1.26, 95%CI: 1.00-1.58, *p*=0.046) (Table 7). While there was no statistical valid after adjusting age and sex.

**Table 7:** *CXCL12* haplotype frequencies and their association with CAD

Haplotype	Freq	case	control	OR (95% CI)	*p*-value
CGCC	0.4943	0.476	0.51	1.00	---
TGCC	0.2131	0.217	0.21	1.10 (0.90 - 1.35)	0.34
CACT	0.1532	0.167	0.141	1.26 (1.00 - 1.58)	0.046
CGTC	0.1158	0.116	0.116	1.06 (0.82 - 1.37)	0.64
CACC	0.0212	0.024	0.019	1.29 (0.75 - 2.21)	0.36

*p* <0.05, statistical significance.

## DISCUSSION

It is widely acknowledged that hereditary factors contribute importantly to the risk of CAD and a large amount of genes and SNPs have been identified to be associated with CAD in various races [15, 16]. In the current study, we detected 6 SNPs to identify the relationship between CAD and *CXCL12* and found three (rs1065297, rs266089 and rs10793538) significant SNPs. As far as we known, these 3 SNPs were first reported to be associated with CAD risk.

GWAS reported that *SDF-1* gene (CXCL-12), which located on 10q11.1 and involved in progenitor cell proliferation, traffic, adhesion and regulates cell survival, associated with cardiovascular disease susceptibility. We also found that rs266089 in *CXCL12* gene increased the CAD risk in total population and in men; rs2839693 in *CXCL12* gene associated with the risk of CAD in men. A study explored the *SDF1* SNPs for prognostic impact in patients with cardiovascular disease, found that cardiovascular who were *SDF1* rs2839693 and rs266089 minor allele carriers showed a significantly better event-free survival probability compared to homozygote carriers of major allele [17]. Apart from this, an article reported that homozygous genotype of the minor allele of rs2839693, A/A, was shown to be significantly decreased in ITP patients, which suggested a protective role of these genotypes [18].

Published studies have shown that the relationship between rs1801157 and CAD susceptibility exist inconsistent. Some studies suggested that rs1801157 in *CXCL12* gene is not associated with the risk of coronary heart disease [19–21]. However, some studies have suggested that *CXCL12* gene may affect the risk of coronary heart disease [22–25]. By integrating the results of other studies, meta-analysis showed that the rs1801157 in *CXCL12* gene was not associated with the risk of coronary heart disease. In our research, we did not find the relationship between SNP rs1801157 and susceptibility to CAD. But, we found that significant differences in TP concentrations with rs1801157 different genotype.

Actually there were some limitations in our study. The sample volume of the case-control study (597 cases and 685 controls) was not relatively large enough. The most defect of our specimen was the mismatching of age and sex. It would still have some random error although in a certain extent though we indeed did some adjustment. The most useful way to solve the question we thought is to take them as independent factors. The main methods of controlling the confounding factors include restrictions on randomization, matching, stratified analysis, multivariate analysis. Due to the age and sex is not match, in the process of statistical analysis, ORs and 95%CIs were computed based on unconditional logistic regression adjusted by age and gender. In addition to, further stratified analysis by sex and age were performed to eliminate the influence of confounding factors.

Our findings in the case-control study provide new evidence for the relationship between SNPs and haplotype of *CXCL12* with CAD risk. What is more, we found different influence of the SNPs between males and females. It's a pity that we did not clarify the mechanism of how the SNPs affect the risk of CAD but we thought it would be a new research direction in the future.

## MATERIALS AND METHODS

### Ethics statement

The protocol in this study conformed to the principles of the Declaration of Helsinki and was ratified by the Ethical Committee of the First Affiliated Hospital of Xi’an Jiaotong University Health Science Center, China. Signed informed consent was obtained from each participant.

### Study participants

A total of 597 patients were recruited in the department of cardiology in First Affiliated Hospital of Xi’an Jiaotong University from. At least two experienced cardiologists performed the diagnosis of CHD according to American Heart Association guidelines [26]. All patients were confirmed by the obstruction of at least 1 large epicardial coronary artery by atheromatous plaque using coronary angiography (>50% diameter stenosis in at least one of the major coronary arteries). Patients who met the exclusion criteria will be excluded from this study: alcohol abuse, diabetes, a history of smoking, chronic lung disease, xanthelasma, and evidence of noncoronary atherosclerotic disease. We also collected the clinical data of CAD patient, including Serum concentrations of ALT, AST, GGT, TP, GLU, TG, TC, HDL-C, LDL-C, APOA, APOB, Lp(a).

Additionally, there were in total 708 relative healthy controls in other departments were involved but 23 were excluded for the defective information or the poor DNA quality. Eventually, 597 case groups and 685 control groups with informed consent were taken into consideration to explore the association between the 6 SNPs and CAD. All the subjects were Chinese Han people and the information was collected by the medical records.

### SNP selection and genotyping

We selected candidate SNPs of *CXCL12* according to previous published papers which demonstrated association with CAD in other ethnic lines and only MAF>5% in the Hapmap Asian population were valid [17]. Finally, a number of 6 SNPs were chosen for further analysis. Genomic DNA extracted and concentrated were accomplished using GoldMag-Mini Purification Kit (GoldMag Co. Ltd. Xian city, China) and spectrometry (DU530 UV/VIS spectrophotometer, Beckman Instruments, Fullerton, CA, USA), respectively. Sequenom MassARRAY RS1000 was applied to genotype the candidate SNPs and subsequently data management and analysis were performed by the Sequenom Typer 4.0 Software [27].

### Statistical analysis

For continuous variable, presented as means ± standard deviations (SDs), performed by T-test; for categorical variables performed by Pearson's chi-square test. From variance analysis assess the differences in continuous variables among the subjects with three genotypes of related genes.

Hardy-Weinberg equilibrium (HWE) was performed for each SNP in control groups using exact test. The differences in allele and genotype frequencies for each SNP between cases and controls were detected using Pearson Chi-Square (χ^2^) test [28]. ORs and 95%CIs were computed based on unconditional logistic regression [29]. Further stratified analysis by sex and age were performed to eliminate the influence of confounding factors. The four genetic models, dominant, co-dominant, recessive and log-additive were applied by PLINK software (http://pngu.mgh.harvard.edu/purcell/plink/) to evaluate the association between the SNPs and CAD.

Statistical analyses were performed using Microsoft Excel and PLINK software. And two-sided *p*-values < 0.05 were considered statistically significant.
